# Optical coherence tomography in neuromyelitis optica spectrum disorders: potential advantages for individualized monitoring of progression and therapy

**DOI:** 10.1007/s13167-017-0123-5

**Published:** 2017-12-22

**Authors:** Frederike C. Oertel, Hanna Zimmermann, Friedemann Paul, Alexander U. Brandt

**Affiliations:** 1 NeuroCure Clinical Research Center, Charité—Universitätsmedizin Berlin, Corporate Member of Freie Universität Berlin, Humboldt-Universität zu Berlin, and Berlin Institute of Health, Charitéplatz 1, 10117 Berlin, Germany; 2 Department of Neurology, Charité—Universitätsmedizin Berlin, corporate member of Freie Universität Berlin, Humboldt-Universität zu Berlin, and Berlin Institute of Health, Berlin, Germany; 3 Experimental and Clinical Research Center, Max-Delbrück-Centrum für Molekulare Medizin und Charité—Universitätsmedizin Berlin, Berlin, Germany

**Keywords:** Neuromyelitis optica, Tomography, optical coherence, Diagnosis, differential, Optic neuritis, Retina, Disease progression, Vision disorders

## Abstract

Neuromyelitis optica spectrum disorders (NMOSD) are mostly relapsing inflammatory disorders of the central nervous system (CNS). Optic neuritis (ON) is the first NMOSD-related clinical event in 55% of the patients, which causes damage to the optic nerve and leads to visual impairment. Retinal optical coherence tomography (OCT) has emerged as a promising method for diagnosis of NMOSD and potential individual monitoring of disease course and severity. OCT not only detects damage to the afferent visual system caused by ON but potentially also NMOSD-specific intraretinal pathology, i.e. astrocytopathy. This article summarizes retinal involvement in NMOSD and reviews OCT methods that could be used now and in the future, for differential diagnosis, for monitoring of disease course, and in clinical trials.

## Neuromyelitis optica spectrum disorders

Neuromyelitis optica spectrum disorders (NMOSD) are autoimmune inflammatory conditions of the central nervous system (CNS) with a mostly relapsing disease course [1]. Clinical hallmarks of NMOSD are optic neuritis (ON), longitudinally extensive transverse myelitis (LETM) in the spinal cord spanning three or more vertebral segments, and brain stem encephalitis including area postrema syndrome [2–5]. Neuropathic pain [6], fatigue [7], and depression [8] are important secondary symptoms. A serum autoantibody against the astrocytic water channel, aquaporin-4 (AQP4-ab), is detectable in approximately 80% of the patients [9–12]. This antibody was shown to be pathogenic, and its detection together with characteristic clinical, epidemiological, and imaging features allows for the discrimination of NMOSD from multiple sclerosis (MS), the most common autoimmune disorder of the CNS and the most relevant differential disease diagnosis [13–17]. NMOSD has distinct immunopathogenesis from MS, which firmly establishes both of these conditions as separate nosologic entities [18–27]. Consequently, disease-modifying treatment differs fundamentally between NMOSD and MS; as many drugs used in MS have proven ineffective or even harmful in NMOSD [28–34]. Conversely, many patients with NMOSD respond well to B cell targeting therapies with rituximab or immunosuppressive therapies with azathioprine or mycophenolate mofetil [29, 33–37]. Recently, an antibody against myelin oligodendrocyte glycoprotein (MOG-ab) was detected in a subgroup of exclusively AQP4-ab seronegative NMOSD patients [38–44], recurrent idiopathic optic neuritis (RION) patients, and a few MS patients [45, 46], further complicating the disease spectrum. Currently, there is controversy over whether these MOG-ab seropositive patients are part of the NMOSD disease spectrum or if they belong to a separate disease entity (“MOG-ab positive encephalomyelitis” or MOG-EM) [47–49]. This article reviews OCT techniques and discusses associations between structural retinal damage and visual function in NMOSD. It will also describe the potential future relevance of OCT for differential diagnosis, patient profiling, individual monitoring of disease course, and for clinical trials with immunosuppressive or potential causal therapies. This article is an updated and extended English version of a recently published article in German [50].

## Optical coherence tomography

### In vivo imaging of the retinal anatomy by OCT

OCT is an interferometric technique employing low-coherent light to produce structural cross-sectional images [51]. The light emitted from the device is backscattered and reflected in a manner dependent on the structural composition of the retina; the interference with a reference beam allows anatomical reconstruction with an axial resolution of a few micrometers (currently approximately 5 μm) [52, 53]. Since its introduction in 1991 by Huang et al. [54], the OCT research has been fast paced. Currently, the most widely used OCT setup is composed of a fixed reference mirror and simultaneous analysis of echoes from all retinal layers by Fourier transformation, thus being called Fourier domain OCT (FD-OCT) or spectral domain OCT (SD-OCT). SD-OCT achieves highly reduced motion artifacts, better reproducibility, and 50 to 100 times faster acquisition than previous methods [55–57]. The novel OCT technology involves the use of a short-cavity swept laser for even higher speed and resolution called swept-source OCT (SS-OCT) [58], as well as the incorporation of volumetric angiography images called optical coherence tomography angiography (OCTA) [59].

**Fig. 1:**
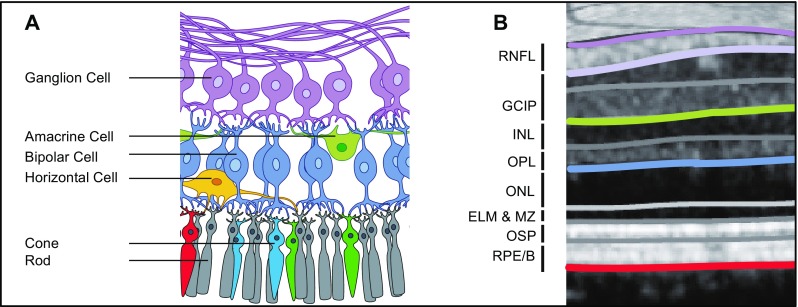
Anatomy of the retina (**a**) with corresponding layers measured by OCT as suggested by Staurenghi et al. [172] and Cruz-Herranz et al. [97] (**b**). Parts of the figure are provided by courtesy of www.neurodial.de [173]. OCT optical coherence tomography, RNFL retinal nerve fiber layer, GCIP combined ganglion cell and inner plexiform layer, INL inner nuclear layer, OPL outer plexiform layer, ONL outer nuclear layer, ELM & MZ external limiting membrane and myoid zone, OSP outer segments of photoreceptors (ellipsoid zone), RPE/B retinal pigment epithelium and Bruch’s complex

Next to MS and NMOSD, a retinal examination by OCT is increasingly applied as a non-invasive technique to evaluate key features of various neurological disorders, e.g., in Susac syndrome, anti-NMDA receptor encephalitis, and also in neurodegenerative diseases [60–65]. OCT provides high-resolution 3D images of retinal structures and can be employed to evaluate the first three neurons of the visual pathway and their interneurons, where one key application is the quantification of neuro-axonal retinal damage (Fig. 1) [66–69].

### Retinal layer thinning and its quantification

The peripapillary retinal nerve fiber layer thickness (pRNFL or sometimes just RNFL or RNFLT) has become a reliable OCT marker for diagnostic evaluation in translational research and care [70–77]. The eponymous retinal nerve fibers are unmyelinated axons of retinal ganglion cells, originating in the retina and leaving the eye through the optic nerve head towards the lateral geniculate nuclei thereby forming the optic nerve. Therefore, these nerve fibers are a suitable model to investigate neuro-axonal damage and neuroprotection in diseases presenting with ON, such as NMOSD, where they are representative of anterograde sections of axons directly affected by ON [78–81]. The pRNFL is measured in ring scans of defined circumference (most commonly 12° or 3.5 mm) around the optic nerve head as mean thickness (in μm) (Fig. 1). By using a ring scan circling the optic nerve head virtually, all axons leaving the eye are included in the measurement, thereby allowing representation of the full axonal content of the respective optic nerve.

The ganglion cell and inner plexiform layer thickness or volume (GCIP or sometimes GCIPL) regularly complements the pRNFL as an imaging marker. The main targets of interest are the very ganglion cell bodies associated with axons in the retinal fiber layers described previously. Due to the poor differentiability in OCT imaging, the ganglion cell layer is usually measured in combination with the adjacent inner plexiform layer as GCIP (Fig. 1). The GCIP is mostly measured as the perifoveal volume (in mm^3^), since the ganglion cells are highly concentrated parafoveally and account for about 34% of the macular volume [78, 82, 83]. Up to 3 months after acute ON when the pRNFL is regularly affected by swelling, the GCIP serves especially well as a stable parameter to quantify retinal neuro-axonal damage [84–87]. Recently, the inner nuclear layer (INL) was suggested to have a swelling specific to an inflammation in autoimmune disorders of the CNS that present with ON [88–91]. The outer retinal layers are currently of lesser interest in neuroinflammatory diseases. Although changes have been described, e.g., after ON or branch retinal artery occlusion in Susac syndrome, high vulnerability to variability from imaging, such as patient positioning and poor reproducibility, makes the interpretation of the outer retinal layer measurements challenging [60, 92, 93].

Retinal measurements between OCT devices from different manufacturers are usually not comparable. Whereas, pRNFL has a reasonably good standardization and is measured similarly across devices, GCIP and INL measurements lack this standardization, thereby impeding comparability [94]. The establishment of standardized criteria for acquisition and assessment of OCT images like the OSCAR-IB criteria for image quality [95, 96] and the APOSTEL reporting guidelines for studies incorporating OCT strive to improve comparability of retinal layer quantification longitudinally, as well as across cohorts [97].

## NMOSD and OCT

### Characteristics of ON as the most common manifestation of NMOSD

ON is the first clinical feature observed in about 55% of the patients with NMOSD and usually causes severe structural damage to the optic nerve and retina with resulting functional impairment [98]. NMOSD patients often suffer from bilateral and sometimes simultaneous ON (radiological bilateral ON: MS ~ 20%, NMOSD ~ 80%), frequent relapses, and severely reduced visual acuity or even complete vision loss [98]. Unilateral ON often appears as afferent pupillary defect (RAPD), while this can be concealed in bilateral ON [99]. Typically, subacute visual loss progresses in the course of days or weeks, and recovery is possible within 6 months since onset [100, 101]. One year after ON, only 52% of the NMOSD patients recover a high contrast visual acuity of 20/20 to 20/63, and about 25% suffer from visual impairment with acuity of < 20/200 [102–104]. Apart from a high contrast visual acuity, patients are often afflicted with severe loss of low contrast visual acuity and decreased vision-related quality of life [105, 106].

### Neuro-axonal damage of the retina after ON

**Fig. 2:**
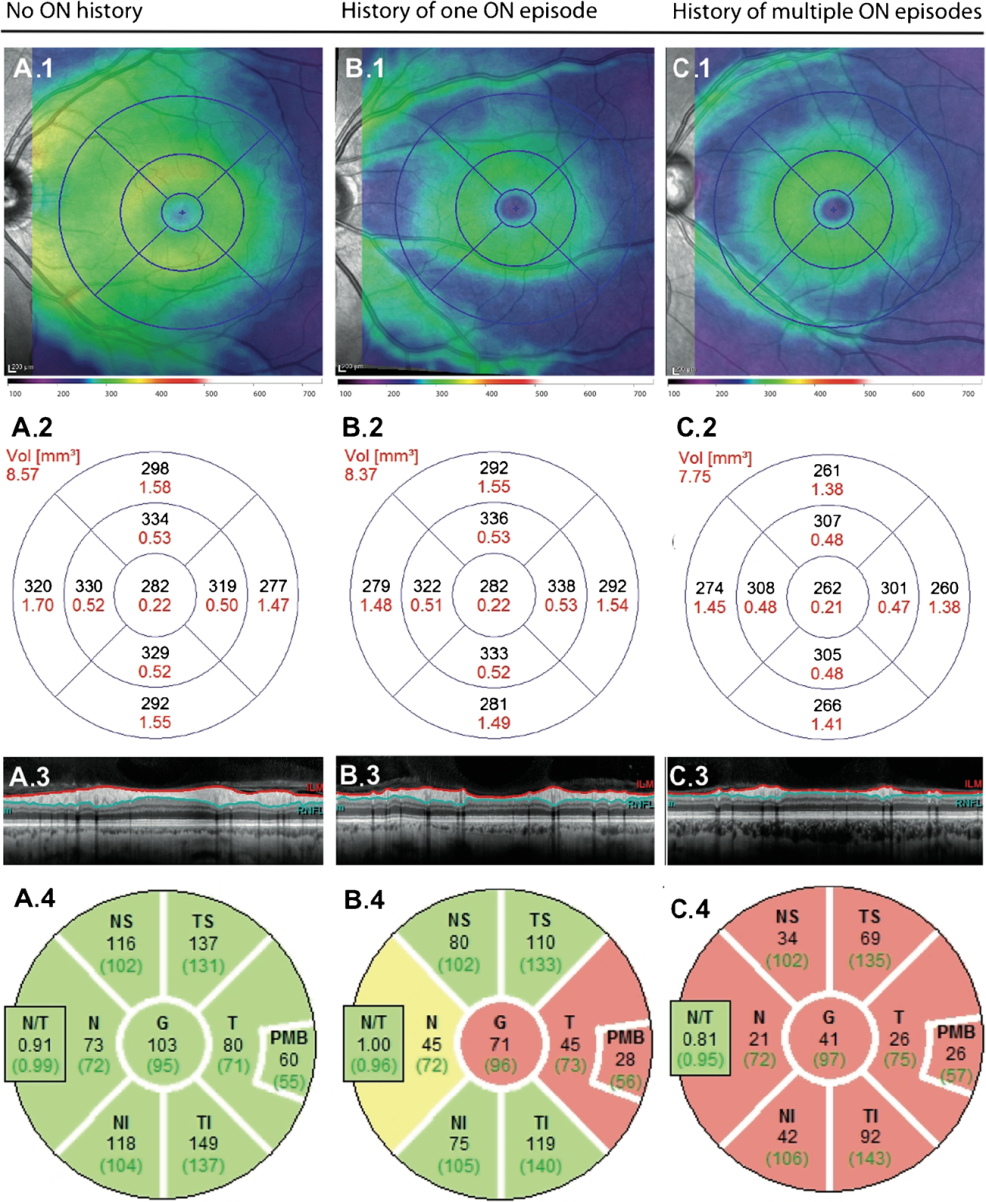
Neuro-axonal damage after ON in NMOSD for **A** an eye not affected by ON in an NMOSD patient compared to **B** an eye after one single ON in an NMOSD patient and **C** an eye after multiple ONs of an NMOSD patient. (1) TMV around the fovea with (2) corresponding macular volume of represented segments. (3) Peripapillary ring scan around the optic nerve head with marked retinal nerve fiber layer for pRNFL measurements. (4) Color-coded image of the pRNFL thicknesses compared to a healthy cohort from the device’s normative database: green: not reduced compared to a healthy cohort (> fifth percentile), yellow: borderline thinned compared to a healthy cohort (< fifth percentile), red: severely reduced compared to a healthy cohort (< first percentile). ON optic neuritis, NMOSD neuromyelitis optica spectrum disorders, pRNFL peripapillary retinal nerve fiber layer, TMV total macular volume

So far, no published studies have investigated acute ON specifically in NMOSD. Studies investigating isolated or idiopathic acute ON, without the distinction of underlying pathologies, have shown that during clinical onset of acute ON, OCT measurements typically give a highly swollen pRNFL that is not representative of retrograde axonal damage [107]. At this time, GCIP thickness is similar in both the affected and the unaffected fellow eye (Table 1) [86]. After acute ON, the loss of retinal axons and ganglion cells proceeds over a period of 6 months [89, 106]. Since the optic nerve is often affected near the chiasm in the AQP4-ab positive NMOSD, potential carryover affects could radiologically or clinically impact the contralateral optic nerve after the unilateral ON [98]. Recurrent ONs in NMOSD give rise to severely thinned pRNFL and GCIP (Fig. 2) [122]. In the case of severe optic nerve atrophy resulting from multiple ON attacks, with pRNFL values lower than 30 μm, further neuro-axonal loss is hard to detect due to flooring effects [99] and the influence of retinal blood vessels running through the measured layers [123]. While retinal damage after ON in MS exhibits a temporal preponderance, all segments can be affected in NMOSD [106, 124]. Pattern variances between NMOSD subtypes are still under investigation: a recent publication suggests a temporal preponderance of retinal damage in MOG-ab seropositive patients as well [111]. Single ONs seem to have less severe effects in MOG-ab seropositive patients compared to AQP4-ab seropositive patients; although the higher frequency of ONs in MOG-ab seropositive patients may result in similar long-range prognoses and may still be unfavorable with respect to visual outcome [119, 125]. After ON, high-contrast visual acuity and low-contrast visual acuity impairment are highly correlated cross-sectionally with reduced pRNFL and GCIP, suggesting both imaging markers as appropriate structural correlates for visual function loss [100, 106, 113, 114]. Relapsing ONs cause pathological latencies of visual evoked potentials (VEP) and severe visual impairment up to complete vision loss [119, 126].

**Table 1:** Most important recent publications on OCT in NMOSD

Reference	Study patients	Controls	Findings
[108]	*N* = 30, 66% AQP4-ab-p.	No	↓ pRNFL only in NMOSD with past ON over 18 months follow-up, independent from relapses
[102]	*N* = 29, 48% AQP4-ab-p., 100% ON, and LETM	*N*(HC) = 45 *N*(LETM only) = 29 *N*(MS-ON) = 29 *N*(MS-NON) = 44	↓ pRNFL in NMOSD vs. all other groups ↓ pRNFL after ON in NMOSD vs. MS ↓ GCIP in NMOSD vs. HC and LETM ↑ INL in NMOSD vs. HC
[109]	*N* = 25, 100% AQP4-ab-p.	No	Microcystic alterations in INL in 15% of the eyes and 24% of the eyes after ON
[110]	*N* = 21, 90% AQP4-ab-p.	*N*(HC) = 34	Time since onset +~ atrophy of gray matter pRNFL +~ pericalcarine cortex thickness
[111]	*N*(AQP4-ab-p.) = 19 *N*(MOG-ab-p.) = 13	*N*(HC) = 13	↓ pRNFL in MOG-ab-p. vs. AQP4-ab-p. NMOSD temporal atrophy in MOG-ab-p. NMOSD
[112]	*N* = 72, 69% ON	*N*(HC) = 34	↓ fovea thickness in NMOSD with and without ON vs. HC; foveal thickness +~ low contrast VA
[113]	*N* = 15, 100% AQP4-ab-p.	*N*(HC) = 23 *N*(MS) = 15	↓ pRNFL, high contrast and low contrast VA in NMOSD vs. MS and HC
[114]	*N* = 33, 100% ON, 52% AQP4-ab-p.	*N*(HC) = 41 *N*(MS) = 60 *N*(LETM) = 28	↓ pRNFL and high contrast VA in NMOSD after ON vs. all other groups ↓ pRNFL in LETM vs. HC
[115]	*N* = 18, 100% ON, 100% AQP4-ab-p.	*N*(MS) = 14	↓ pRNFL in NMOSD vs. MS pRNFL +~ high contrast VA pRNFL −~ number of attacks and −~ time until high-dose corticosteroid treatment
[116]	*N* = 31, 71% ON, 100% AQP4-ab-p.	*N*(HC) = 34	↓ foveal thickness and FA in NMOSD with and without ON vs. HC ↓ pRNFL in NMOSD only after ON vs. HC
[50]	*N* = 40, 92, 5% AQP4-ab-p.	No	Vessel artifacts in pRNFL measurements −~ pRNFL
[72]	*N* = 23, 70% ON, 56% AQP4-ab-p.	*N*(HC) = 75 *N*(MS) = 110	= pRNFL in NMOSD and MS after ON ↓ temporal pRNFL without ON in MS vs. NMOSD ↓ pRNFL and GCIP in NMOSD without ON vs. HC
[117]	*N* = 9, 100% ON, 67% AQP4-ab-p.	No	No RNFL or macular thinning observed over 4 years follow-up
[118]	*N* = 22, 77% ON, 100% AQP4-ab-p.	*N*(MS) = 47	↓ pRNFL after ON in NMOSD vs. to MS More severe superior and inferior affection in NMOSD
[119]	*N*(AQP4-ab p.) = 16 *N*(MOG-ab-p.) = 16	*N*(HC) = 16	↓ pRNFL, GCIP, high contrast VA in AQP4-Ak-p., and MOG-ab-p. NMOSD vs. HC = structural and functional parameters in AQP4-ab-p. vs. MOG-ab-p. NMOSD ↑ ON rate in MOG-ab-p. vs. AQP4-ab-p. NMOSD
[120]	*N* = 26, 100% ON, 60% AQP4-ab-p.	*N*(HC) = 77 *N*(MS) = 378 *N*(LETM) = 17	↓ pRNFL and TMV after ON in NMOSD vs. to MS = pRNFL and TMV in non-ON NMOSD eyes and HC
[105]	*N* = 31, 74% ON, 65% AQP4-ab-p.	*N*(MS) = 31	↓ vision-related quality of life in NMOSD vs. MS vision-related quality of life +~ high contrast and low contrast VA and pRNFL and GCIP
[106]	*N* = 17, 60% ON, 94% AQP4-ab-p.	*N*(HC) = 17 *N*(MS) = 17	↓ pRNFL, GCIP and low contrast VA in NMOSD vs. HC ↑ INL and outer retinal layers in NMOSD after ON vs. NMOSD without ON, MS, and HC
[89]	*N* = 39	*N*(HC) = 39	↓ pRNFL, GCIP, outer retinal layers, and low contrast VA in NMOSD vs. HC microcystic INL alterations in 26% of the NMOSD patients (after ON only)
[121]	*N*(MOG-ab-p) = 6	*N*(AQP4-ab-p.) = 10	↓ pRNFL and VA after ON in AQP4-ab-p. vs. MOG-ab-p. NMOSD
[86]	*N* = 22, 73% AQP4-ab-p.	*N*(HC vs. NMOSD) = 22 *N*(HC vs. MS) = 50 *N*(MS) = 98 *N*(acute ON) = 20	↓ pRNFL, GCIP, and TMV after ON vs. without ON in NMOSD and MS ↓ GCIP in non-ON NMOSD vs. HC

*N* number, *vs.* versus, *↓* reduction, *↑* increase, *+~* positive correlation, *-~* negative correlation, *pRNFL* peripapillary retinal nerve fiber layer, *GCIP* combined ganglion cell and inner plexiform layer, *INL* inner nuclear layer, *ON* optic neuritis, *AQP4-ab-p*. aquaporin-4 antibody positive, *FA* fractional anisotropy, *HC* healthy controls, *LETM* longitudinally extensive transverse myelitis, *MS* multiple sclerosis, *MOG-ab-p*. myelin oligodendrocyte glycoprotein antibody positive, *VA* visual acuity

### Primary retinal pathology in NMOSD

**Fig. 3:**
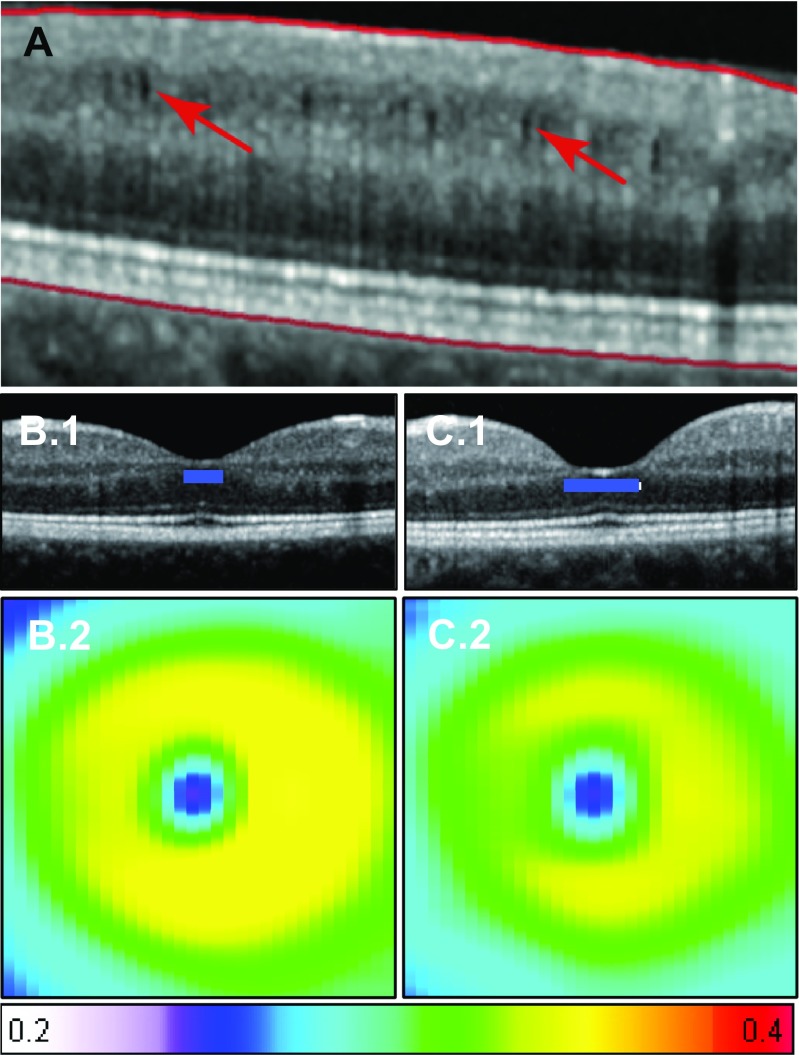
Primary retinal pathology in NMOSD. **A** Macular microcysts in the INL of a NMOSD after ON (arrows: microcysts). **B** (1) OCT and (2) mean shape surface reconstruction with shape variation (color code: thickness in mm + 1 SD) of healthy cohort compared to **C** (1) OCT and (2) mean shape surface reconstruction with shape variation of broadened fovea surface in a NMOSD cohort. ON optic neuritis, NMOSD neuromyelitis optica spectrum disorders, INL inner nuclear layer, SD standard deviation

Around 20% of the NMOSD patients have microcystic alterations of the INL after ON (Fig. 3) [89, 90, 109, 127]. This so-called microcystic macular edema (MME) is characteristic for a range of optic neuropathies and is not specific for NMOSD. It has also been reported, although not as frequently, in MS patients with ON and from patients with non-inflammatory optic neuropathies [90, 128, 129]. Its formation in NMOSD seems to be dynamic and caused through intraretinal processes, although vitreous traction might play an additional role in some cases [127, 130]. The detailed pathology of MME is not yet clear; possible explanations include vascular damage with extracellular fluid accumulation, the aforementioned vitreous traction and Mueller cell pathology [128, 127, 131].

Mueller cells are astrocytic cells of the retina residing mainly in the INL and might also play a role in NMOSD beyond MMO and ON-inflicted damage. They have multiple responsibilities, including water homeostasis, energy metabolism, and neurotransmitter recycling. Since they express AQP4 water channel proteins, they might be a direct target of AQP4-ab and a potential cause of a primary retinopathy in NMOSD [132–134]. Animal studies and human autopsy reports support the concept of NMOSD as a primary astrocytopathy. In one study, a retinal damage model in rats showed complement-independent loss of AQP4 in Mueller cells [132]. Autopsies of the afferent visual system demonstrated complement-independent loss of retinal Müller cells also in humans [135]. This is further substantiated by in vivo data from the fovea in NMOSD, where Mueller cells reside in high concentration. In AQP4-ab seropositive NMOSD, the foveal and parafoveal regions are thinned while the pRNFL and the GCIP seem to be unaffected in patients without a history of ON (Fig. 3) [112, 116]. The presumed primary retinopathy could potentially enable a quicker diagnosis and sensible tracking of disease course in the future, but research in this regard is still lacking [136, 137]. While a recent study by Tian et al. [138] found that there is also retinal neuro-axonal damage without ON in NMOSD, longitudinal studies investigating neuro-axonal damage without ON in NMOSD have not been performed extensively. The only two studies published so far have shown conflicting results, where Bouyon et al. [108] showed RNFL thinning over 18 months in patients with a past ON, but Manogaran et al. [117] were not able to show RNFL or macular thinning over a 4-year follow-up. Thus, further longitudinal studies are required, to investigate the development of retinal damage in NMOSD beyond ON and their potential functional relevance.

### Association between OCT and magnetic resonance imaging

The magnetic resonance imaging (MRI) of brain and spinal cord is an indispensable tool and a part of the diagnostic criteria for MS and NMOSD [4, 139–144]. In NMOSD, the association between brain tissue alterations and intraretinal or afferent visual system changes is not completely understood. Retrograde and anterograde trans-synaptic degeneration following ON potentially causes subsequent alterations in the retina, optic nerve, and anatomically connected tracts [145–148]. Consequently, a combination of lesion length in the optic nerve measured by MRI and retinal findings by OCT offers the unique possibility of predicting visual outcome after ON [125]. Recently, a study with a mixed AQP4-ab seropositive and seronegative NMOSD cohort that had cortical atrophy showed a correlation between pRNFL and pericalcarine cortex thickness, further supporting the concept of trans-synaptic degeneration being responsible for some detectable brain atrophy [110]. Also, intracerebral changes are accentuated in the optic radiation and can consequently be understood as ON-associated transmitted damage [149]. Nevertheless, a functional MRI study by Finke et al. suggests that not only are there degenerative processes that contribute to impaired vision in NMOSD but maladaptive plasticity after ON may also play a role [150].

While numerous studies exist describing brain tissue alterations in MS (global atrophy, atrophy of grey and white matter, microstructural changes by diffusion-weighted imaging (DWI)), only few studies have investigated MRI characteristics in NMOSD [149, 151–154]. The existence of diffuse tissue alterations with global or regional atrophy in NMOSD is therefore still a matter of debate [155, 156]. Up to 80% of the AQP4-ab seropositive NMOSD patients present with cerebral lesions in AQP4-rich sites like the hypothalamus and periependymal regions; where up to 15% would formally fulfil the diagnostic criteria for MS [157, 158]. In contrast to MS, cortical lesions are rare in NMOSD [159, 160]. Joint analyses of diffusion tensor imaging of the optic radiation and OCT data from AQP4-ab seropositive patients suggest microstructural damage of the afferent visual system also in patients without a history of ON, supporting diffuse brain changes detectable by MRI outside of trans-synaptic degeneration [116, 138]. In line with this, a study from Ventura et al. showed a spinal cord atrophy in patients without LETM and spinal cord lesions, which points towards an attack-independent tissue damage in the spinal cord [161]. Ultimately, the latter three studies included only a few patients and further studies investigating attack-independent tissue alterations in NMOSD with higher sample sizes and in different anatomical regions are highly warranted.

### The relevance of OCT for clinical trials in NMOSD

To date, no results from randomized controlled trials (RCTs) of disease-modifying therapies (DMTs) in NMOSD have been published. Current treatment strategies (e.g., rituximab, azathioprine, mycophenolate mofetil, oral prednisolone, recently also tocilizumab) are based on retrospective case series or uncontrolled trials [29, 30, 33–37]. Importantly, DMTs used in MS (e.g., beta-interferon, glatiramer acetate, natalizumab, fingolimod, alemtuzumab) are ineffective in NMOSD patients or can even provoke relapses [31, 32, 162]. Therefore, the development of safe and effective DMTs for NMOSD is highly warranted [163, 164]. Several RCTs in this regard are currently conducted or planned [165–167]. In these and future trials, OCT may serve as a valuable outcome parameter to evaluate the structural sequelae of ON attacks or to track subclinical retinal changes. To date, multiple RCTs in MS and ON have successfully used OCT measures like pRNFL as primary or secondary endpoints [73, 168, 169]. In NMOSD, smaller retrospective studies evaluating the effect of therapies based on OCT parameters were performed that suggest the superiority of the combination of plasmapheresis and corticosteroid therapy compared to corticosteroid therapy alone and confirmed a preserving effect on RNFL of early high-dose methyl prednisolone therapy in acute ON [113, 115]. Future RCTs in NMOSD may incorporate the predictive value of structural OCT parameters for visual function in parallel to common clinical endpoints, such as pRNFL and GCIP as markers of neuro-axonal damage and INL as a marker for inflammation [84, 170].

## Outlook

The retina is one of the most affected CNS regions in NMOSD. The OCT is an easy-to-use diagnostic tool to assess neuroinflammatory and neurodegenerative processes in the retina and thus the visual system. An early examination of the retina by OCT in NMOSD might provide useful information on the severity of structural damage that may be predictive of functional outcomes, as well as in the long-term disease course [171]. With regard to NMOSD-specific pathology, OCT measurements can also provide key information for differential diagnosis against other disease entities. In the future, OCT might also help to evaluate the success of NMOSD-specific therapies. Adequately powered studies investigating longitudinal changes both after ON in NMOSD but also outside ON are currently lacking and should be a priority of future research.

## Abbreviations

AQP4-ab: aquaporin-4 antibodies
CNS: central nervous system
DMT: disease modifying therapy
DWI: diffusion-weight imaging
ELM: external limiting membrane
FA: fractional anisotropy
FD-OCT: Fourier domain optical coherence tomography
GCIP: combined ganglion cell and inner plexiform layer
HC: healthy control
INL: inner nuclear layer
LETM: longitudinally extensive transverse myelitis
MMO: microcystic macular edema
MOG-ab: myelin-oligodendrocyte glycoprotein antibody
MOG-EM: MOG-Enzephalomyelitis
MRI: magnet resonance imaging
MS: multiple sclerosis
MZ: myoid zone
NMOSD: neuromyelitis optica spectrum disorders
OCT: optical coherence tomography
OCTA: optical coherence tomography angiography
ON: optic neuritis
ONL: outer nuclear layer
OPL: outer plexiform layer
OSP: outer segments of photoreceptors
pRNFL: peripapillary retinal nerve fiber layer
RAPD: relative afferent pupillary defect
RCT: randomized controlled trial
RION: relapsing isolated optic neuritis
RNFL: retinal nerve fiber layer
RPE/B: retinal pigment epithelium and Bruch’s membrane complex
SD: standard deviation
SD-OCT: spectral-domain optical coherence tomography
SS-OCT: swept-source optical coherence tomography
TMV: total macular volume
VA: visual acuity
VEP: visual evoked potential
